# Up‐regulation of HDACs, a harbinger of uraemic endothelial dysfunction, is prevented by defibrotide

**DOI:** 10.1111/jcmm.14865

**Published:** 2019-11-28

**Authors:** Marta Palomo, Manel Vera, Susana Martin, Sergi Torramadé‐Moix, Julia Martinez‐Sanchez, Ana Belen Moreno, Enric Carreras, Ginés Escolar, Aleix Cases, Maribel Díaz‐Ricart

**Affiliations:** ^1^ Hematopathology Centre Diagnòstic Biomèdic (CDB) Hospital Clinic Institut d'Investigacions Biomèdiques August Pi i Sunyer (IDIBAPS) Universitat de Barcelona (UB) Barcelona Spain; ^2^ Josep Carreras Leukaemia Research Institute Hospital Clinic/University of Barcelona Campus Barcelona Spain; ^3^ Barcelona Endothelium Team (BET) Barcelona Spain; ^4^ Nephrology Department Hospital Clinic Institut d'Investigacions Biomèdiques August Pi i Sunyer (IDIBAPS) Universitat de Barcelona (UB) Barcelona Spain

**Keywords:** chronic kidney disease, defibrotide, endothelial dysfunction, HDAC, HDAC1, HDAC2, inflammation, oxidative stress

## Abstract

Endothelial dysfunction is an earlier contributor to the development of atherosclerosis in chronic kidney disease (CKD), in which the role of epigenetic triggers cannot be ruled out. Endothelial protective strategies, such as defibrotide (DF), may be useful in this scenario. We evaluated changes induced by CKD on endothelial cell proteome and explored the effect of DF and the mechanisms involved. Human umbilical cord vein endothelial cells were exposed to sera from healthy donors (n = 20) and patients with end‐stage renal disease on haemodialysis (n = 20). Differential protein expression was investigated by using a proteomic approach, Western blot and immunofluorescence. HDAC1 and HDAC2 overexpression was detected. Increased HDAC1 expression occurred at both cytoplasm and nucleus. These effects were dose‐dependently inhibited by DF. Both the HDACs inhibitor trichostatin A and DF prevented the up‐regulation of the endothelial dysfunction markers induced by the uraemic milieu: intercellular adhesion molecule‐1, surface Toll‐like receptor‐4, von Willebrand Factor and reactive oxygen species. Moreover, DF down‐regulated HDACs expression through the PI3/AKT signalling pathway. HDACs appear as key modulators of the CKD‐induced endothelial dysfunction as specific blockade by trichostatin A or by DF prevents endothelial dysfunction responses to the CKD insult. Moreover, DF exerts its endothelial protective effect by inhibiting HDAC up‐regulation likely** **through PI3K/AKT.

## 1 INTRODUCTION

Chronic kidney disease (CKD) is a major public health concern due to its increased prevalence, high morbidity and mortality associated, high costs, poorer quality of life and reduced life expectancy.^1^ CKD is associated with an increased cardiovascular (CV) risk, and CV disease accounts for nearly half of all deaths.^2^ The increased incidence of CV disease (CVD) in CKD cannot be explained by the high prevalence of traditional risk factors in this setting, and classical CV risk scores, such as the Framingham risk score, underestimate the CV risk in this population.^3^ Further, the results of pharmacological interventions that have proven a cardio‐protective benefit in the general population or other high risk populations (eg in diabetes or in patients with established cardiovascular disease), such as statins or inhibitors of the renin‐angiotensin system, have failed to show a benefit in patients with end‐stage renal disease.^4^, ^5^, ^6^, ^7^ Because of that, new or uraemia‐related CV risk factors have been postulated to play a role in the CV risk of these patients.^8^ Thus, there is a need for the search of new therapies able to reduce the high CV risk and the associated mortality in this population.

There is wide evidence indicating that endothelial dysfunction is an initial stage for the development of atherosclerosis, and in vivo and in vitro studies have demonstrated that endothelial dysfunction is present in CKD.^9^ In previous in vitro studies, endothelial dysfunction induced by the CKD milieu has been characterized. Exposure of endothelial cells (ECs) to sera from CKD patients results in morphological alterations, enhanced thrombogenicity of the extracellular matrix and changes towards a pro‐inflammatory phenotype, with increased expression of adhesion receptors and activation of signalling pathways, such as nuclear factor‐kappa B (NFkB),^10^ or the innate immunity Toll‐like receptor 4 (TLR4) and the NALP3 inflammasome.^11^ Moreover, the endothelial response to the CKD insult is also characterized by an enhanced oxidative stress and changes in the expression of related proteins.^12^, ^13^, ^14^, ^15^

Our previous in vitro results suggest that the CKD setting induces activation of specific genes in the endothelium that promotes a prooxidant, pro‐inflammatory, prothrombotic and proproliferative state. In this regard, HDACs enzymes are important epigenetic factors that regulate pro‐inflammatory gene expression, in ECs and in other tissues, and also deacetylate non‐histone proteins that regulate inflammatory signalling.^16^ Therefore, they appear to be promising therapeutic targets for the treatment of atherosclerosis and other cardiovascular diseases.^17^, ^18^ Furthermore, HDAC activity is linked to a number of cardio‐renal pathologies, including heart failure,^19^ hypertension,^20^ and diabetes or diabetic kidney disease.^21^, ^22^ Thus, the dysfunctional endothelium and its epigenetic regulation arise as an attractive target for interventions designed to reduce the risk and burden of CVD in CKD patients.^23^, ^24^ Defibrotide (Defitelio®) (DF) is a drug composed by a complex single‐stranded oligodeoxyribonucleotides derived from porcine intestinal mucosal DNA that has demonstrated profibrinolytic, antithrombotic‐thrombolytic, antiischaemic, antishock, antiatherosclerotic, antirejection and anti‐angiogenic effects.^25^ The endothelial protective activity of this drug has been demonstrated in different clinical settings.^26^, ^27^, ^28^ This drug interacts specifically with the membrane of ECs where it displays its anti‐inflammatory and anti‐oxidant effects.^29^

In the present study, we applied a translational approach^12^ to identify proteins differentially expressed in an endothelium exposed to the CKD milieu and the effect of DF. Functional assays were performed to confirm the role of the most physiologically relevant identified proteins and their implication in the development of endothelial dysfunction. Moreover, we aimed at identifying the mechanisms involved in the protective effect exerted by DF in this setting.

## 2 METHODS

### 2.1 Patients and sample collection

Sera from patients with end‐stage renal disease on maintenance haemodialysis (HD, n = 20) were included. Patient characteristics are detailed in Table 1. In order to exclude causes other than uraemia in endothelial dysfunction, patients with diabetes, dyslipidaemia and/or previous CVD and/or current smokers were excluded. All CKD patients were dialysed with biocompatible membranes, ultrapure water, for ≥4 hours, three times a week with a Kt/V ≥ 1.3. None of the patients were dialysed through an HD catheter as a vascular access. Blood samples were collected before the 2nd or 3rd HD session of the week. Sex and age‐matched healthy donors (n = 20), with no previous history of CVD, and preserved renal function (eGFR > 90 mL/min/1.73 m^2^, CKD‐EPI formula) were enrolled as controls (Table 1). Serum samples from CKD patients and from healthy donors were obtained by centrifugation of non‐anticoagulated blood (3000 *g*, 15 minutes) and stored at −80°C until used. Pools of serum were prepared containing serum samples from different healthy donors or HD patients, which were used as reagent for the different experiments performed.

**Table 1:** Clinical characteristics of patients and controls

	Control (n = 20)	HD (n = 20)
Age, years, mean ± SD	48.3 ± 9.3	55.4 ± 15.7
Female/male	12/8	9/11
Estimated filtration rate CKD‐EPI (mL/min/1.73 m^2^)	>90	0
Mean time on dialysis, months ± SD		23 ± 5.7
Dialysis adequacy, Kt/V, mean, ±SD		2.3 ± 0.4
Serum albumin (g/L), mean ± SD	44 ± 4	39 ± 6
CRP (mg/dL), mean ± SD	0.2 ± 0.1	0.4 ± 0.2
Haemoglobin (g/L), mean ± SD	12.6 ± 1.6	11.3 ± 0.4
Leucocytes (10^9^/L), mean ± SD	7.4 ± 1.6	6.8 ± 1.2
Causes of CKD n (%)		
Glomerulonephritis		4 (20%)
Polycystic kidney disease		3 (15%)
Interstitial nephropathy		4 (20%)
Obstructive kidney disease		2 (10%)
Nephrosclerosis		3 (15%)
Unknown		4 (20%)
Hypertension n (%)	0	14 (70%)
Use of statins n (%)	0	8 (40%)
Use of vitamin D n (%)	0	18 (90%)
Use of erythropoiesis stimulating agents n (%)	0	17 (85%)
Use of inhibitors of the renin‐angiotensin system n (%)	0	6 (30%)

Abbreviation: CRP, C‐Reactive protein.

### 2.2 Endothelial cell cultures

ECs were isolated from human umbilical cord veins.^30^ Cells were maintained and subcultured at 37°C in a 5% CO_2_ humidified incubator in EGM‐2 Endothelial Cell Growth Medium‐2 BulletKit (Lonza, Cultek SLU). Culture medium was replaced every 48 hours. To perform the experiments, ECs were used between the 2nd and 3rd passage. To study the CKD‐induced endothelial damage, we used an established in vitro model of uraemic endothelial dysfunction.^11^, ^13^, ^14^, ^15^, ^24^ This model consists of exposing endothelial cells to Medium 199 (Gibco BRL, Life Technologies) supplemented with 100 U/mL penicillin, 100 g/mL streptomycin (Gibco BRL, Life Technologies) and 20% of pooled sera from patients (n = 20) or from matched healthy donors (n = 20), for different periods depending on the endothelial damage marker to be evaluated.

### 2.3 Differential proteomic analysis

ECs were isolated from human umbilical cord veins (n = 6) and cultured as previously described. After reaching confluence during the first passage, cells were mixed, seeded in 75‐cm^2^ flasks and exposed to media containing 20% of pooled sera of controls (n = 20 distributed in 6 different pools) or CKD patients (n = 20 distributed in 6 different pools) with or without DF (100 μg/mL, a dose selected from our previous studies)^27^, ^29^ during 24 hours. Then ECs were washed, trypsinized, counted, pelleted and stored at –80°C until proteomic analysis.

Each sample was then solubilized in Laemmli lysis buffer (60 mmol/L Tris HCl, pH 6.8, 2.2% (w/v) SDS, 5% (v/v) glycerol, 0.1 mol/L DTT), incubated for 3 minutes at 99°C, sonicated and centrifuged for 15 minutes. Protein concentration was determined using the Quick Start Bradford Protein Assay (Bio‐Rad) following the manufacturer's recommendation.^31^ Then, 100 µg of proteins was separated by using SDS‐PAGE 12% acrylamide gel. The gel was fixed with 30% ethanol and 10% acetic acid and preconditioned with sodium thiosulphate, before silver nitrate staining treatment with the modified Blum et al^32^ silver staining protocol. Gels were cut into 3 mm slices prior to trypsin digestion and peptide extraction.^33^ Peptides were separated by means of nano liquid chromatography using a Proxeon EASY‐nLC (Thermo Fisher Scientific) with a flow rate of 300 nL/min, a C18 trap column (5 µm, 120 Å, 100 µm inner diameter 2 cm in length), an EASY C18 analytical column (3 µm, 120 Å, 75 µm inner diameter 10 cm in length). The following linear gradient, using solvent B (97% acetonitrile, 0.1% formic acid) and solvent A (3% acetonitrile, 0.1% formic acid), was employed: 5%‐40% buffer B (90 minutes); 40%‐100% buffer B (5 minutes); 100% buffer B (15 minutes) finally. MS/MS analysis was performed using an LTQ Orbitrap Velos (Thermo Fisher Scientific) coupled to a nanoelectrospray ion source (Thermo Fisher Scientific). MS/MS data acquisition was completed using Xcalibur 2.1 (Thermo Fisher Scientific).^34^

Data were processed using Proteome Discoverer 1.2 (Thermo Fisher Scientific). For database searching, processed data were submitted to the in‐house Homo sapiens UniProtKB/Swiss‐Prot database (released June 2011; 20 211 protein entries) using SEQUEST, version 28.0 (Thermo Fisher Scientific). The following search parameters were used: two maximum missed cleavages for trypsin; carbamidomethylation as a fixed modification; methionine oxidation as a variable modification; 20 pp peptide mass tolerance; and 0.8 Da fragment ion tolerance. Criteria used to accept identification included a false discovery rate (FDR) of 0.05, and five minimum spectra (and at least one unique peptide) matched per protein. Proteins identified were classified according to biological function(s) using the information available at the UniProt Knowledge base (UniProtKB/Swiss‐Prot) website (http://www.uniprot.org) and checked according to previous descriptions found in PubMed (http://www.ncbi.nlm.nih.gov/pubmed/; National Center for Biotechnology Information, US National Library of Medicine, National Institutes of Health, Bethesda, MD). Spectral counts were normalized using the average of the sum of raw counts across all samples. Differentially expressed proteins were identified using stringent selection criteria: fold change ≥3 for up‐regulated proteins, detected in all samples and a mean normalized count in positive samples of ≥2.

### 2.4 Confirmation of proteins by Western blot and immunofluorescence studies

From the differentially up‐regulated proteins found comparing the analysed settings, both HDAC1 and HDAC2 were selected to be deeply studied. First, protein lysates from ECs exposed to the same conditions studied in the proteomic assay were analysed by Western blot (WB, n = 4) and immunofluorescence (IF, n = 6) with specific primary antibodies (Abcam) to confirm the proteomic results.

For WB analysis, ECs were exposed to media containing 20% of pooled sera of controls (4 different pools) or CKD patients (4 different pools) with or without DF (100 μg/mL, a dose selected from our previous studies)^27^, ^29^ during 24 hours. Then, ECs were lysed with Laemmli's buffer, sonicated to shear DNA and reduce viscosity (15 seconds), and heated to 90°C (5 minutes). Protein concentration in the supernatants was determined using Coomassie Plus (Pierce). Samples were resolved by 8% SDS‐PAGE, proteins transferred to nitrocellulose membranes and probed with specific antibodies against HDAC1, HDAC2 (Abcam) and B‐actin (Cell Signaling). Membranes were incubated with a peroxidase‐conjugated anti‐rabbit IgG and developed by chemiluminescence.^29^ Densitometric assay of protein bands was performed using Fiji (ImageJ, National Institutes of Health), and all values were normalized with their respective B‐actin bands.

For IF analysis, ECs were exposed to the same conditions previously described, fixed with 4% paraformaldehyde in 0.15 mol/L PBS, pH 7.4 (4°C, 10 minutes), blocked with 1% BSA, permeabilized with triton X‐100 (Sigma) and incubated with a primary antibody against HDAC1 and a secondary antibody anti‐rabbit IgG conjugated with Alexa 488 (Molecular probes). Nuclei were stained with DAPI (Sigma). Samples were evaluated by a fluorescence microscopy (Leica DM4000B), images were captured through a video camera (Leica DFC310FX), and the percentage of the area positive for staining was calculated by computerized establishing a threshold through morphometric analysis (Fiji, ImageJ, National Institutes of Health). Moreover, with the aim of analysing the location of HDAC1, a colour threshold tool defining blue colour was used to differentiate nuclei (blue) and cytoplasm (not blue). Then, HDAC1 expression was differentially quantified in these two compartments. Moreover, to evaluate the specificity of HDAC1 inhibition by DF, a dose‐dependent study (0, 50 and 100 µg/mL) was performed by using an IF assay.

### 2.5 Roles of HDAC1 and HDAC2 in endothelial damage induced by CKD sera

To evaluate the role of HDAC1 and HDAC2 in uraemic endothelial dysfunction, a pan‐HDAC inhibitor trichostatin A (TSA) (Sigma Aldrich) and DF were used as HDAC inhibitors. ECs on 6‐well microplates were pretreated (24 hours) with TSA (50 nmol/L) or DF (100 µg/mL) and exposed to media containing 20% of pooled sera from the uraemic patients or healthy donors, as previously described for the differential proteomic analysis. Cells were then fixed and labelled for intercellular adhesion molecule‐1 (ICAM‐1) (Millipore, Merck), Toll‐like receptor 4 (TLR4) (Abcam) and von Willebrand Factor (vWF) (DAKO) using IF, as previously described. Reactive oxygen species (ROS) production was explored by using CM‐H2DCFDA. This compound can passively diffuse into cells being oxidized by ROS to the highly fluorescent dichlorofluorescein. ROS production was monitored by fluorescence microscopy. All samples were evaluated by microscopy (Leica DM4000B), and images were captured through a videocamera (Leica DFC310FX). Fluorescence micrographs were analysed using ImageJ (version 1.43m; NIH, http://rsb.info.nih.gov/nih-image/manual/tech.html#). Cultured cells were selected from the background with the threshold tool, and the fluorescence intensity was measured.

### 2.6 Activation of PI 3‐kinase/AKT pathway as inducer of HDAC1 and HDAC2 expression and inhibitory effect of defibrotide

740 Y‐P (Tocris Bioscience, Bio‐Techne R&D Systems, S.LU), a cell‐permeable PI 3‐kinase/AKT activator, was used as reagent to investigate the interaction between DF and HDAC1 and HDAC2. 740 Y‐P was added (20 μmol/L, 5 hours) to ECs cultures in the presence or absence of DF and HDAC1 and HDAC2 expression was evaluated through IF (n = 6) and WB (n = 4).

### 2.7 Study approval

Written informed consent was obtained from all participants, and the study was approved by the Ethical Committee of the Hospital Clinic (HCB/2014/0302) and carried out according to the principles of World Medical Association Declaration of Helsinki.

### 2.8 Statistics

Results are expressed as mean ± standard error of the mean (SEM) and correspond to the % of labelled area/% of nuclei area for IF assays when analysing total HDAC1 expression, in % of labelled area when analysing HDAC1 expression in nuclei or cytoplasm, and in fold increase vs control for the values obtained from densitometric analysis of the protein bands normalized with their respective B‐actin bands for WB. N corresponds to different experiments performed with different pools (from healthy donors or CKD patients) for each value analysed. Statistical analysis was performed using SPSS statistical package 17.0.0 (SPSS Inc, Chicago, IL) with raw data using *t* test. Results were considered statistically significant when *P* < .05.

## 3 RESULTS

### 3.1 Endothelial dysfunction in CKD is characterized by an increase in HDAC1 and HDAC2, among other proteins

To study the proteomic changes involved in endothelial uraemic dysfunction and the potential protective role of DF in this setting, a proteomic approach was performed on ECs exposed to sera of healthy controls or CKD patients in the absence and presence of DF. After normalization, 11 proteins were differentially expressed comparing the analysed settings (Table 2). Proteins related to inflammation, such as protein arginine N‐methyltransferase 5 or histone arginine methyltransferase; proteins involved in epigenetic regulation and proteins related to cell motility; proliferation and survival (HDAC1, HDAC2, ATP‐dependent DNA helicase Q1, TRIO and F‐acting‐binding protein), among others were overexpressed in the uraemic setting. Results suggested that DF could re‐establish the proper expression of proteins that were found overexpressed in the ECs exposed to CKD conditions.

**Table 2:** Overexpressed proteins in endothelial cells exposed to CKD sera

Swiss‐Prot accession no.	Protein name	Gene	Cellular location	Molecular function	Biological process
P33121	Long‐chain‐fatty‐acid‐CoA ligase 1	ACSL1	Mitochondrion, endoplasmic reticulum, and peroxisome	Ligase, activation of long‐chain fatty acids for both synthesis of cellular lipids, and degradation via beta‐oxidation	Fatty acid metabolism, lipid metabolism
O14744	Protein arginine N‐methyltransferase 5	PRMT5	Golgi apparatus, nucleus, and cytoplasm	Chromatin regulator methyltransferase, repressor, and transferase	Biological rhythms, transcription
Q03701	CCAAT/enhancer‐binding protein zeta	CEBPZ	Nucleus	Activator	Transcription regulation
Q9UJW0	Dynactin subunit 4	DCTN4	Cytoskeleton, cell cortex, and sarcomere	Protein N‐terminus binding	Endoplasmic reticulum to Golgi vesicle‐mediated transport and nuclear migration
Q9BY44	Eukaryotic translation initiation factor 2A	EIF2A	Cytosol, extracellular region, and secreted	Initiation factor	Protein biosynthesis and translation regulation
Q92556	Engulfment and cell motility protein 1	ELMO1	Plasma membrane	SH3 domain binding	Actin cytoskeleton organization, apoptotic process, and vascular endothelial growth factor receptor signalling pathway
Q13451	Peptidyl‐prolyl cis‐trans isomerase FKBP5	FKBP5	Nucleus and cytoplasm	Heat shock protein binding	Chaperone‐mediated protein folding
Q96I24	Far upstream element‐binding protein 3	FUBP3	Nucleus	DNA‐binding transcription activator activity, RNA polymerase II‐specific, and RNA binding	Positive regulation of gene expression
Q96PP9	Guanylate‐binding protein 4	GBP4	Golgi apparatus and nucleus	GTPase activity	Cellular response to interferon‐gamma
Q13547	Histone deacetylase 1	HDAC1	Nucleus	Chromatin regulator, Hydrolase, and repressor	Biological rhythms and Transcription regulation
Q92769	Histone deacetylase 2	HDAC2	Nucleus and cytoplasm	Chromatin regulator, hydrolase, and repressor	Biological rhythms and transcription regulation

### 3.2 DF prevents the overexpression of HDAC1 and HDAC2 induced by CKD sera, as well as increased HDAC1 in both nucleic and cytoplasmic locations

**Figure 1:**
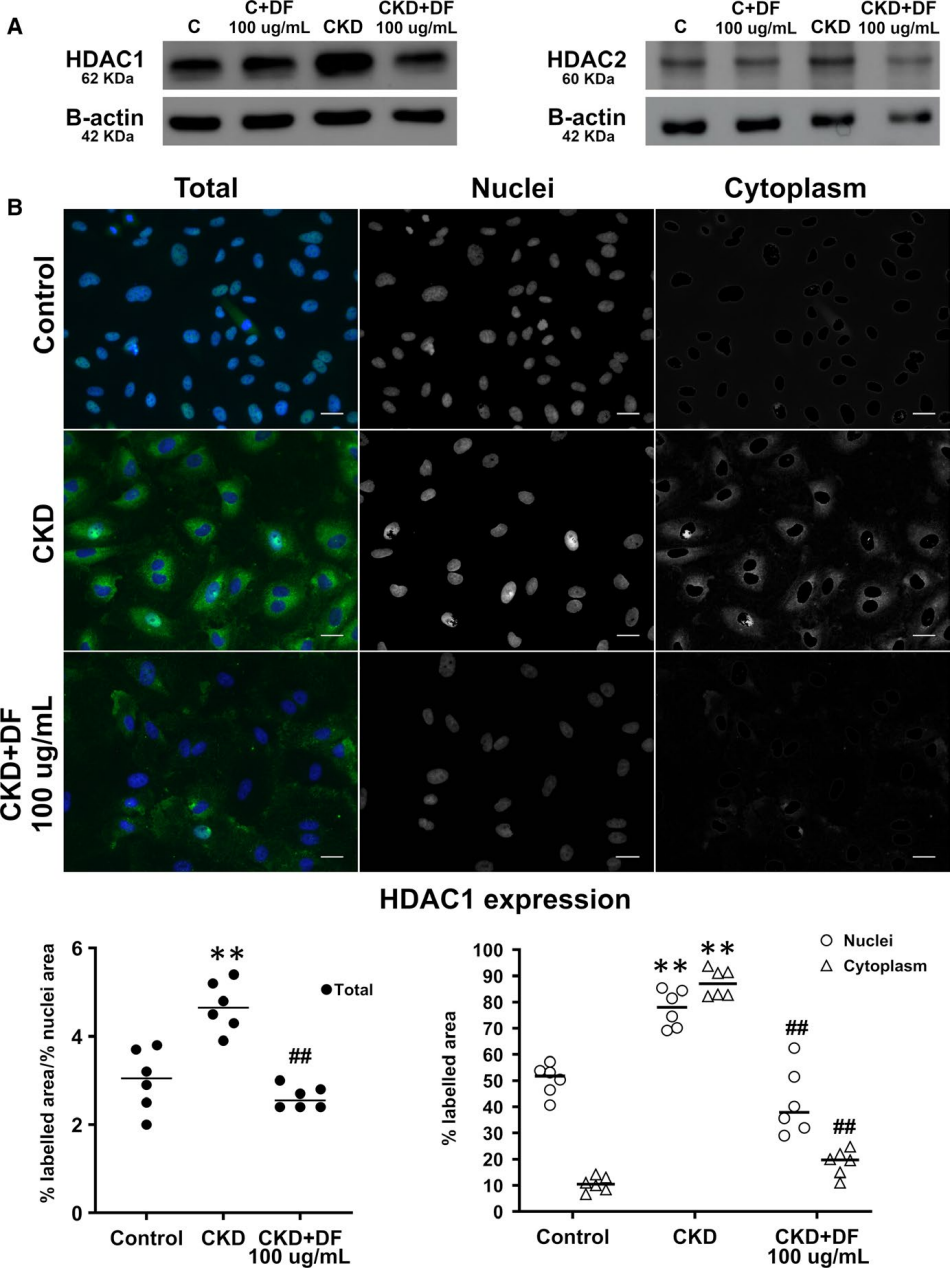
Defibrotide prevents HDAC1 increased expression induced by CKD sera at both nucleic and cytoplasmatic locations. A, Immunoblot images show expression of HDAC1 (left) and HDAC2 (right) when endothelial cells were exposed to control or CKD sera (24 h) in the absence or presence of DF (100 µg/mL). B, Micrographs show HDAC1 expression (green) in endothelial cells exposed to control and CKD sera in the absence or presence of defibrotide (CKD + DF). Left column shows total presence of HDAC1 (green) and nucleic (blue) staining. The middle column shows HDAC1 expression only in the nuclei and the right column shows HDAC1 expression only in the cytoplasm (40× magnification). Left scatterplot (with median) represents HDAC1 expression in terms of the total percentage of labelled area/percentage of nuclei area, and right scatterplot represent HDAC1 expression in terms of the total percentage of labelled area in nuclei or cytoplasm, in endothelial cells exposed to healthy sera (Control) and sera from CKD patients with or without the presence of defibrotide (CKD and CKD + DF, respectively) (n = 6, being **P < .01 vs control and ##*P* < .01 vs CKD, n = number of independent experiments, statistical analysis was performed with raw data using *t* test)

WB and IF techniques were applied to confirm HDAC1 and HDAC2 overexpression in ECs exposed to CKD sera and the effect of DF. WB results confirmed that CKD induced an overexpression of HDAC1 (1.9 ± 0.3 fold vs Control, n = 4, *P* < .05) and HDAC2 (1.5 ± 0.1 fold vs Control, n = 4, *P* < .05) that was normalized in the presence of DF (1.1 ± 0.2 for HDAC1, and 0.9 ± 0.2 for HDAC2, n = 4 and *P* < .05 vs CKD for all experiments) (Panel A, Figure 1). Similarly, an increase in HDAC1 was detected by IF in ECs exposed to CKD from 3 ± 0.3% of labelled area/% nuclei area in controls to 4.7 ± 0.2% of labelled area/% nuclei area in CKD (n = 6, *P* < .01). The effect of uraemic sera on endothelial HDAC1 expression was prevented by DF (2.6 ± 0.1% of labelled area/% nuclei area, n = 6, *P* < .01 vs CKD) (Micrographs and left scatterplot, Panel B, Figure 1). Specifically at the nucleus, CKD sera induced a significant increase in HDAC1 (77.5 ± 2.9% vs 50.3 ± 2.4% of nuclei labelled area in control samples, n = 6, *P* < .01), which was prevented by DF (41.8 ± 5.2% of nuclei labelled area, n = 6, *P* < .01 vs CKD). In addition, the effect of DF was also notable in the cytoplasmic location in ECs exposed to CKD, as the overexpression of the protein in ECs exposed to uraemic sera vs controls was reduced to almost 75% of cytoplasmic labelled area (Micrographs and right scatterplot, Panel B, Figure 1).

### 3.3 The inhibitory effect of DF on CKD‐induced HDAC1 overexpression is dose‐dependent

**Figure 2:**
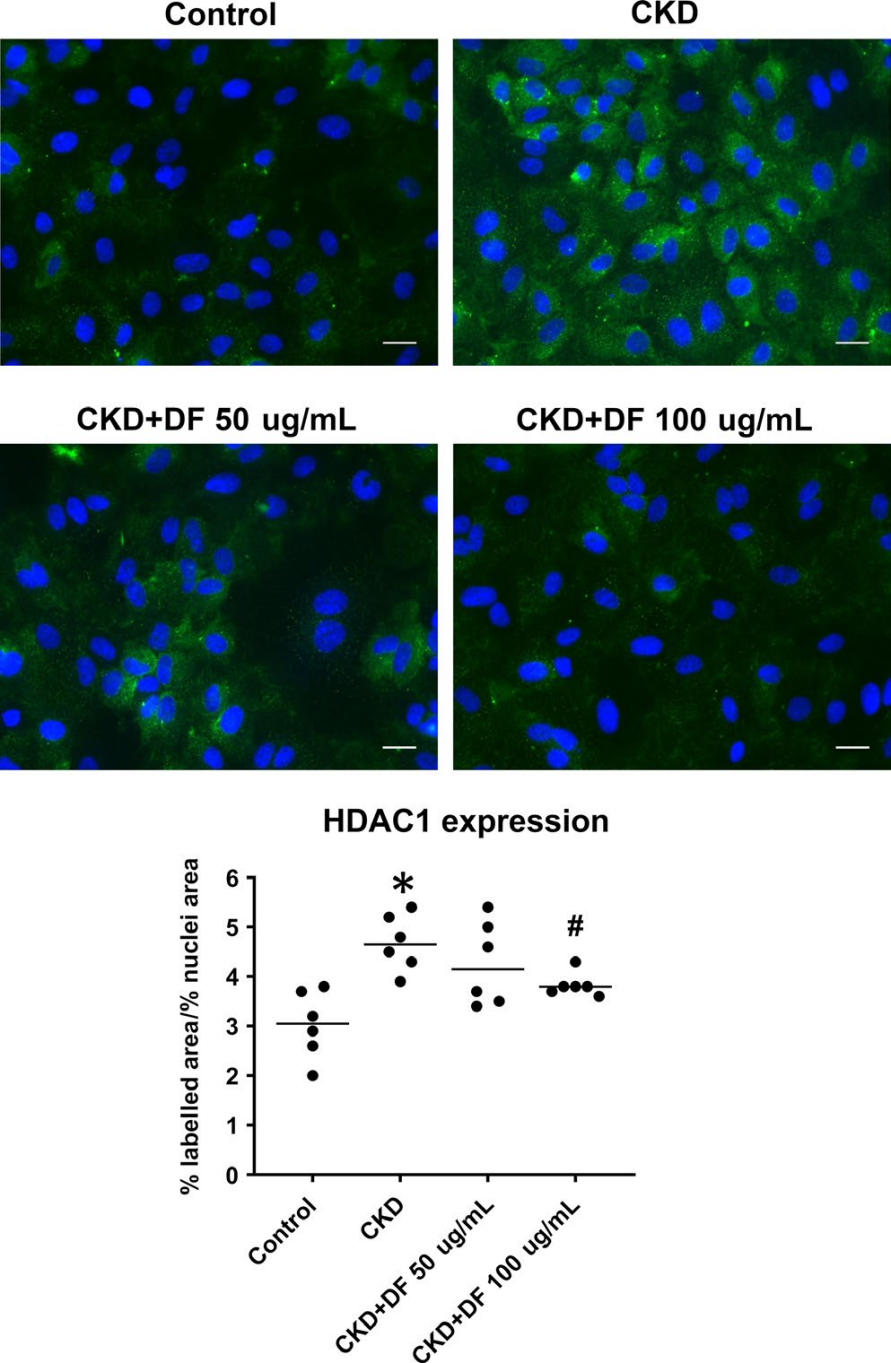
Defibrotide prevents HDAC1 increased expression induced by CKD sera in a dose‐dependent manner. Micrographs show endothelial cells exposed to CKD sera in absence (up) and presence of defibrotide (50 µg/mL and 100 µg/mL). Nuclei are labelled with DAPI (blue) (40× magnification). Scatterplot (with median) represents quantification of the area positive for HDAC1 staining normalized by percentage of nuclei area, by immunofluorescence technique (n = 6, being *P < .05 vs control and #*P* < .05 vs CKD, n = number of independent experiments, statistical analysis was performed with raw data using *t* test)

Immunofluorescence assays were performed with different DF doses to test the specificity of the reduction of HDAC1 expression previously detected. In ECs exposed to CKD sera, HDAC1 total expression increased to 4.7 ± 0.2% of labelled area/% nuclei area compared to control, and was dose‐dependently inhibited in the presence of 50 μg/mL (4.2 ± 0.3% of labelled area/% nuclei area, n = 6,) and 100 μg/mL (3.8 ± 0.1% of labelled area/% nuclei area, n = 6, *P* < .05 vs CKD) of DF (Figure 2).

### 3.4 CKD‐induced endothelial dysfunction is mediated through HDAC1 and HDAC2 overexpression

**Figure 3:**
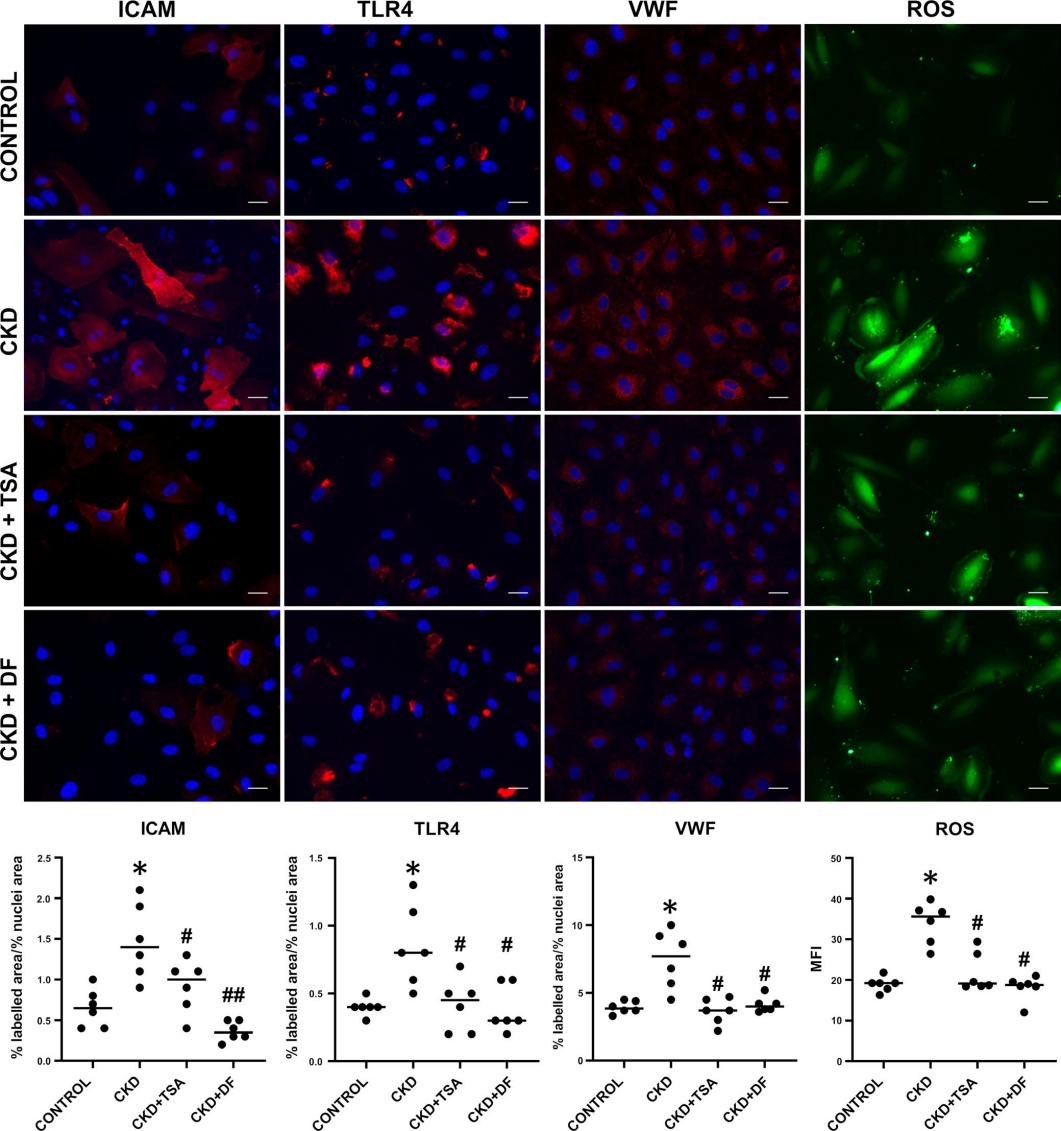
ICAM‐1, TLR4, vWF and ROS increases in CKD are mediated through HDACs induction. Micrographs show enhanced expression of the adhesion receptor ICAM‐1 (red), TLR4 (red), the adhesive protein vWF (red) and production of ROS (green) when endothelial cells were exposed to CKD patients' sera. The co‐incubation of cells with CKD patients sera and TSA (CKD + TSA, middle row) or defibrotide (CKD + DF, lower row) induced a decrease in the expression of the markers of endothelial damage analysed. Cell nuclei are stained with DAPI (blue) (40× magnification). Scatterplots (with median) correspond to the quantification of the endothelial damage markers evaluated. All data correspond to relative expression compared to control levels (n = 6, being *P < .05 vs control and #*P* < .05 and ##*P* < .01 vs CKD, n = number of independent experiments, statistical analysis was performed with raw data using *t* test)

ICAM‐1 and TLR4 expression on cell surfaces and vWF content were higher in ECs exposed to the CKD patients’ sera when compared to control sera (1.5 ± 0.2%, 0.8 ± 0.1%, and 7.5 ± 0.9% vs 0.6 ± 0.1%, 0.4 ± 0.1%, and 3.9 ± 0.2%, respectively, n = 6, *P* < .05). Exposure of ECs to CKD sera in the presence or absence of TSA was performed to assess if HDAC1 and HDAC2 overexpression mediated the increases in markers of endothelial damage induced by CKD. Our results point out that inhibition of HDACs overexpression reduced the increase in the markers of endothelial damage evaluated. In the presence of TSA, ICAM‐1 expression was reduced from 1.5 ± 0.2% to 0.9 ± 0.1%, TLR4 from 0.8 ± 0.1% to 0.4 ± 0.1%, vWF from 7.5 ± 0.9% to 3.6 ± 0.4% and ROS production from 4.1 ± 2.1 of mean fluorescence intensity to 21.8 ± 2.0% (*P* < .05 for all values) (Figure 3). Furthermore, DF was also capable to inhibit the expression of ICAM‐1, TLR4 and vWF content on cells exposed to uraemic sera in a significant manner, decreasing their expression to control levels (0.4 ± 0.1%, 0.4 ± 0.1% and 4.1 ± 0.2%, respectively, n = 6, *P* < .05 vs CKD for ICAM and *P* < .05 vs CKD for TLR4 and vWF) (Figure 3). DF also exhibited a remarkably and significant inhibitory effect on ROS generation in response to the uraemic sera, reducing its production to control levels (18.1 ± 1.3 of mean fluorescence intensity, n = 6, *P* < .05 vs CKD) (Figure 3).

### 3.5 Effect of DF on HDAC1 and HDAC2 is potentially mediated through PI3K/AKT pathway inhibition

**Figure 4:**
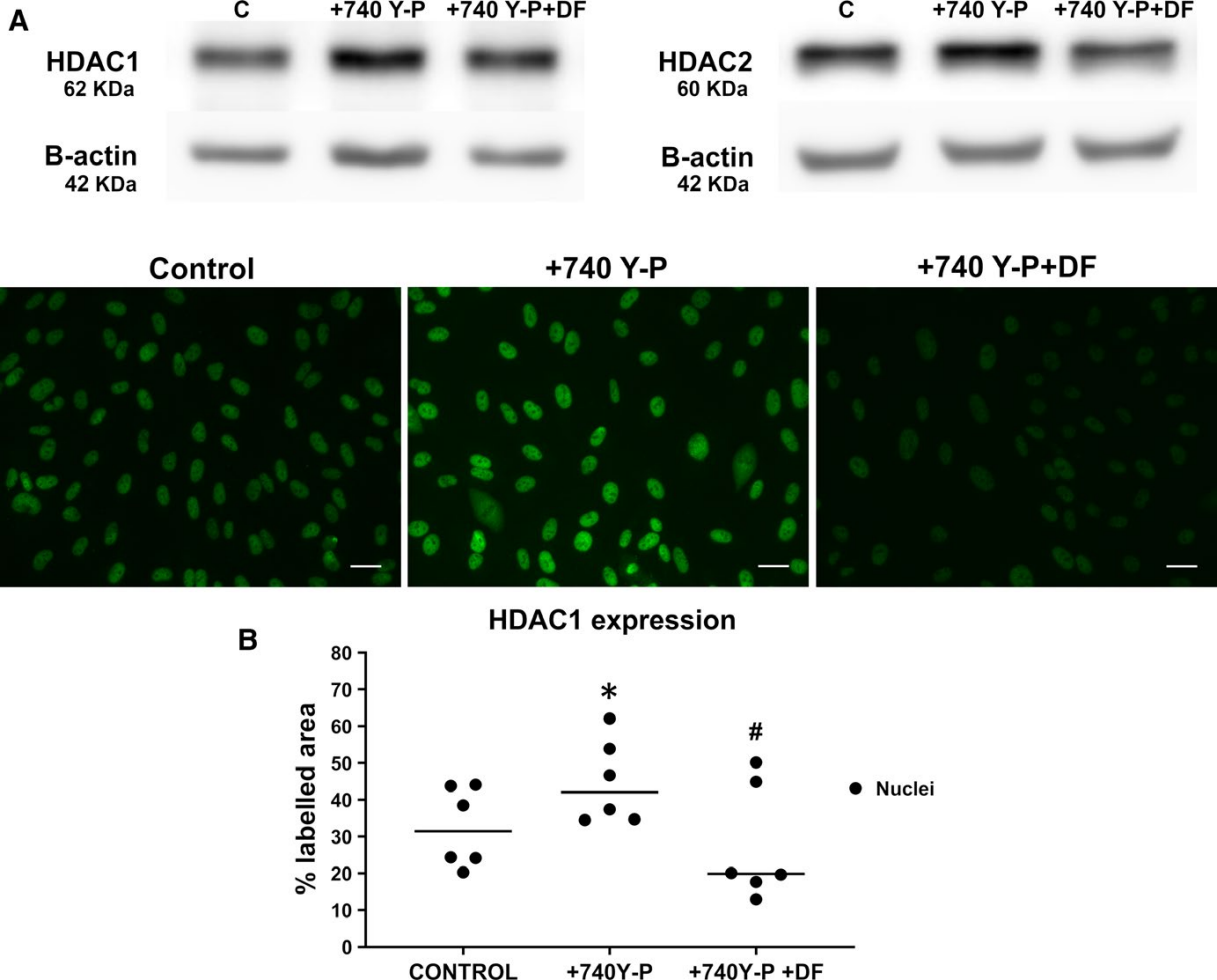
Defibrotide acts as a PI3/AKT inhibitor to interact with HDACs. A, Immunoblot images show expression of HDAC1 (left) and HDAC2 (right) when endothelial cells were exposed to 740 Y‐P in absence or presence of DF (100 µg/mL). B, Micrographs show an increase in HDAC1 expression (green) in endothelial cells exposed to P740‐Y‐P (+P740‐Y‐P) and a decrease when DF was added (+740 Y‐P + DF). Scatterplot (with median) represents the quantification of HDAC1 expression in the three situations (Control, +740 Y‐P, +740 Y‐P + DF) in terms of the labelled area (n = 6, being *P < .05 vs control and #*P* < .05 vs CKD, n = number of independent experiments, statistical analysis was performed with raw data using *t* test)

ECs were exposed to P740‐Y‐P, a cell‐permeable phosphopeptide activator of the PI3K/AKT pathway in the presence or absence of DF (100 μg/mL). Then, HDAC1 expression was assessed by WB and IF, and HDAC2 by WB (Figure 4).

WB results revealed that the expression of HDAC1 and HDAC2 was increased in ECs incubated with P740‐Y‐P (5 hours) (fold of 1.9 ± 0.1 and 1.4 ± 0.2, respectively vs control, n = 4, *P* < .05) and that these increases were prevented by DF (0.9 ± 0.1 and 1.1 ± 0.2 fold vs control, respectively). Moreover, through an IF assay HDAC1 overexpression in the nuclei was confirmed after the incubation of ECs with P740‐Y‐P (from 32.6 ± 4.4% of covered area to 44.9 ± 6.4%, n = 6, *P* < .05). Defibrotide was able to prevent this increase (27.6 ± 5.9, n = 6, *P* < .05).

## 4 DISCUSSION

**Figure 5:**
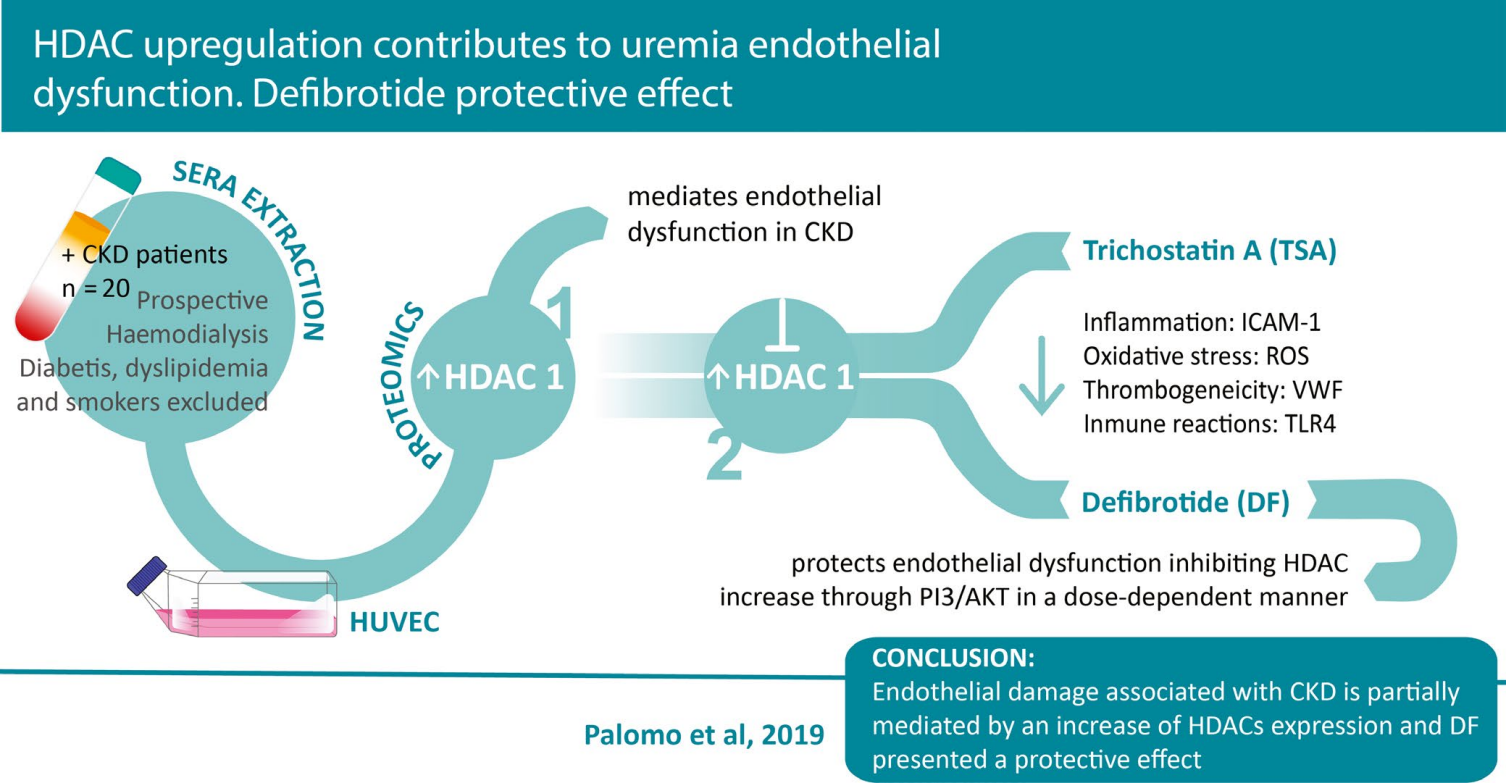
Visual abstract. A single, concise, pictorial and visual summary of the main findings of the present study in which we demonstrate that HDACs appear as key modulators of the CKD‐induced endothelial dysfunction and that DF prevents endothelial dysfunction responses to the CKD insult likely through PI3K/AKT

Our present study explored the protein signature of the endothelium exposed to CKD sera in the presence and absence of DF, and pointed out to HDACs as key molecules that mediate the endothelial response to the CKD milieu. Both TSA and DF prevented the endothelium from developing its pro‐inflammatory, prooxidant, prothrombotic and activated innate immunity phenotype induced by the CKD sera. Further, PI3K/AKT signalling pathway was identified as a putative pathway through which DF modulates HDACs expression (Figure 5). Thus, the results of the present study highlight the relevance of the epigenetic changes associated with endothelial dysfunction in CKD and uncover the potential mechanisms of action by which DF exerts its protective effect on ECs in this setting.

The involvement of vascular endothelium in the initiation and the progression of atherosclerosis in CKD patients has been progressively recognized. So far, the endothelial phenotype in CKD has been extensively characterized, but there is a lack of information regarding the mechanisms through which the uraemic milieu exerts an impact on the endothelial cell and its epigenome. To approach this knowledge, we applied an established translational methodology^12^ to look for the signature of endothelial dysfunction in CKD and find key factors that may be susceptible to be regulated by DF. We were able to identify two proteins, HDAC1 and HDAC2, involved in epigenetic regulation, among other up‐regulated proteins. Epigenetics refer to chromatin‐based mechanisms important in the regulation of gene expression that do not involve changes in the DNA sequence per se.^35^, ^36^

Our proteomic results were confirmed by other techniques and revealed that the CKD milieu induces overexpression of both HDAC1 and 2 and the accumulation of HDAC1 in both the nucleus and the cytoplasm of ECs. These two enzymes are widely expressed in human tissues, belong to class I HDACs, which are considered the ‘classical’ HDACs, whose activities could be inhibited by TSA, and are involved in cell proliferation and cell survival.^37^ Although HDAC class I (HDAC 1, 2, 3 and 8) is classically described as being expressed in the nucleus, these proteins are also found in the cytosol. In this sense, several studies have shown that HDAC1 can also be expressed in the cytosol in different cell lines,^38^, ^39^, ^40^ and although its expression is mainly located in the nucleus, its expression is enhanced in the cytosol in pathological conditions.^41^ Further, it has been reported that the up‐regulation of HDAC1 in ECs deacetylates nitric oxide synthase 3 (NOS_3_), directly resulting in a decrease of endothelial nitric oxide production.^42^ HDAC overexpression in ECs in response to CKD sera is in concordance with previous evidence by other authors showing enhanced expression of these proteins in other cardio‐renal pathologies.^19^, ^20^, ^21^, ^22^ The protective effect of DF in this setting concord with its proved beneficial effect in other clinical entities associated with endothelial dysfunction.^27^, ^28^, ^29^

HDACs positively act on pro‐inflammatory candidates, such as cytokines (IL‐6, IL‐12, TNF), chemokines (CCL2, CCL7 and CXCL) and other inflammatory mediators (MMP‐9 and endothelin‐1).^43^, ^44^ In this regard, HDACs inhibitors show broad anti‐inflammatory effects through different mechanisms. In our model of uraemic endothelium, both TSA and DF reduced ICAM‐1 expression and vWF production. Moreover, there are many studies demonstrating the important role of HDACs both in innate and adaptive immunity. In particular, these enzymes seem to act on the expression of TLR target genes.^45^ In this regard, we show in the present study that TLR4 expression is regulated by HDACs in ECs exposed to a uraemic milieu. In addition, the inhibition of ROS production by TSA and DF demonstrates that HDACs are also involved in the prooxidant endothelial response to CKD.

The pharmacological inhibition of HDACs is a new target for several disorders. These drugs have been approved for the treatment of some lymphomas, or epilepsy (valproic acid) and several others are in clinical trials. Further, these agents have shown its efficacy in animal models of inflammatory diseases, attenuating the progression of renal fibrogenesis in obstructive nephropathy or reducing cyst formation in polycystic kidney disease.^46^ This property has been successfully applied to ameliorate endothelial activation and vascular pathophysiology in sickle cell anaemia in transgenic mice. In this experimental animal model, and also in HUVECs,^47^, ^48^ TSA significantly reduced VCAM‐1 and tissue factor expression.^49^ Although many of the underlying mechanisms and functions of HDACs in human cells are poorly understood and remain unclear, they appear to be promising targets in the modulation of endothelial dysfunction.

Once we demonstrated that DF is able to reduce HDACs expression and their downstream effects in the CKD‐induced endothelial damage, we focused on the search of the signalling pathway potentially used by the drug to modulate these epigenetic regulators. There is evidence demonstrating that HDAC expression is regulated by the PI3K/AKT pathway.^17^, ^50^ In addition, our group has previously demonstrated that DF inhibits the activation of PI3K/AKT.^27^, ^51^ Both facts prompted us to hypothesize that DF was regulating HDACs through this pathway. To prove this hypothesis, ECs were exposed to a PI3K/AKT inducer to stimulate HDAC expression, and then, the effect of DF was explored. Our results indicate that DF could act as a PI3K/AKT pathway inhibitor to modulate HDACs expression. One of the limitations of our study is that we measured protein levels, but not HDACs activity or other HDACs not appearing in our proteomic approach. Another limitation of our study is that we used a pan‐HDAC inhibitor rather than specific HDAC1 or 2 inhibitor, and its effects on other HDACs cannot be ruled out.

In conclusion, CKD is an example of a complex disease in which the phenotype arises from a combination of environmental and inheritable factors.^52^ Evidence suggests that the contribution of the uraemic milieu may be mediated via modifications of the epigenome. The findings of the present study revealed a role for HDACs as key modulators of the endothelial phenotype in response to the CKD insult. These enzymes seem to mediate, at least in part, the endothelial enhanced oxidative stress, the up‐regulation of the innate immunity, and the pro‐inflammatory and pro‐thrombogenic responses. In addition, we provide strong evidence showing that DF may confer a protection to the endothelium from the uraemic insult acting as a potential HDAC modulator. Finally, DF seems to exert its endothelial protective effect by inhibiting HDAC up‐regulation likely through PI3K/AKT signalling pathway.

## CONFLICT OF INTEREST

MDR and MP declares conflict of interest with Jazz Pharmaceuticals plc/Gentium Inc in the form of speaker's fee for symposiums, and EC declares conflict of interest with Jazz Pharmaceuticals plc/Gentium Inc as consultant and in the form of speaker's fee for symposiums. All other authors have declared that no conflict of interest exists.

## AUTHORS CONTRIBUTIONS

MP designed and conducted the experiments, analysed the results, designed the images and wrote the manuscript. MV selected the patients, performed the images and reviewed the results and the manuscript. SM contributed to the design and the conducting of the experiments, acquired the data and analysed the results. ST and JMS contributed to the conduction of the experiments and the maintenance of cell cultures. ABM, EC, GE, and AC reviewed the results and the manuscript. MDR. designed and supervised the project and reviewed the results and the manuscript.

## Data Availability

The data that support the findings of this study are available from the corresponding author upon reasonable request.
